# Crystal structure of methyl 4-(2-fluoro­phenyl)-6-methyl-2-sulfanylidene-1,2,3,4-tetra­hydro­pyrimidine-5-carb­oxy­late

**DOI:** 10.1107/S2056989015018873

**Published:** 2015-10-14

**Authors:** M. S. Krishnamurthy, Noor Shahina Begum

**Affiliations:** a Department of Studies in Chemistry, Bangalore University, Bangalore 560 001, Karnataka, India

**Keywords:** crystal structure, pyrimidine derivative, hydrogen bonding,C—H⋯π inter­actions

## Abstract

In the title compound, C_13_H_13_FN_2_O_2_S, the pyrimidine ring adopts a twist-boat conformation with the MeC*N* and methine-*C* atoms displaced by 0.0938 (6) and 0.2739 (3) Å, respectively, from the mean plane through the other four atoms of the ring. The 2-fluoro­benzene ring is positioned axially and forms a dihedral angle of 89.13 (4)° with the mean plane through the pyrimidine ring. The crystal structure features N—H⋯O, N—H⋯S and C—H⋯O hydrogen bonds that link mol­ecules into supra­molecular chains along the *b* axis. These chains are linked into a layer parallel to (10-1) by C—H⋯π inter­actions; layers stack with no specific inter­actions between them.

## Related literature   

For the bioactivity of organo-fluorine compounds, see: Guru Row, (1999 ▸); Yamazaki *et al.*, (2009 ▸). For biological activity of pyrimidine derivatives, see: Kappe (2000 ▸) and of di­hydro­pyrimidines (DHPMs) and their derivatives, see; Jauk *et al.* (2000 ▸); Kappe (1998 ▸); Mayer *et al.* (1999 ▸). For the Biginelli reaction, see: Biginelli (1893 ▸). For bond length data, see: Qin *et al.* (2006 ▸). For related structures, see: Krishnamurthy & Begum (2015*a* ▸,*b* ▸).
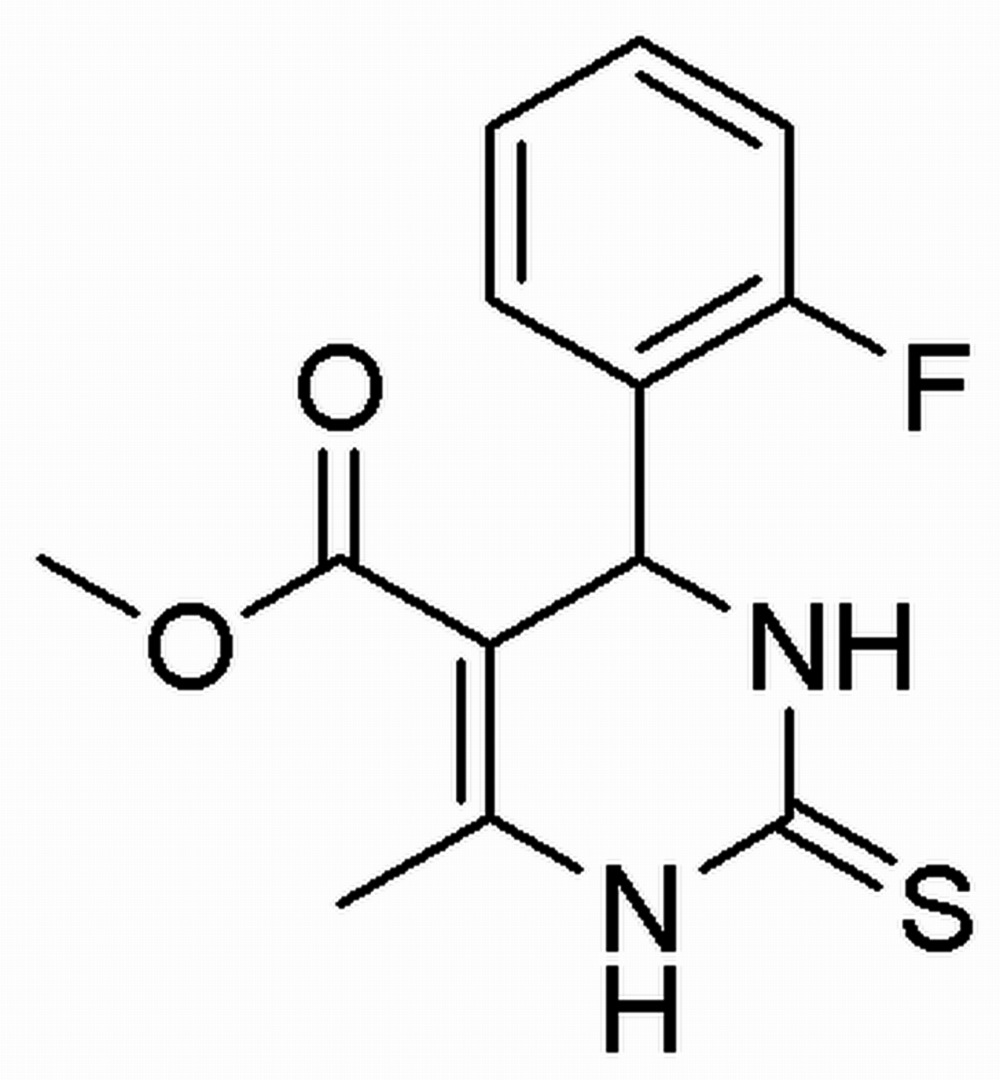


## Experimental   

### Crystal data  

C_13_H_13_FN_2_O_2_S
*M* *_r_* = 280.31
Monoclinic, 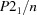

*a* = 13.3298 (15) Å
*b* = 7.1509 (8) Å
*c* = 14.5703 (17) Å
β = 109.854 (4)°
*V* = 1306.3 (3) Å^3^
*Z* = 4
Mo *K*α radiation
μ = 0.26 mm^−1^
*T* = 100 K
0.24 × 0.22 × 0.18 mm

### Data collection  

Bruker SMART APEX CCD diffractometer
Absorption correction: multi-scan (*SADABS*; Bruker, 1998 ▸) *T*_min_ = 0.955, *T*_max_ = 0.960
9937 measured reflections
2296 independent reflections
1340 reflections with *I* > 2σ(*I*)
*R*_int_ = 0.112

### Refinement  

*R*[*F* ^2^ > 2σ(*F* ^2^)] = 0.064
*wR*(*F* ^2^) = 0.133
*S* = 0.95
2296 reflections
174 parameters
H-atom parameters constrained
Δρ_max_ = 0.42 e Å^−3^
Δρ_min_ = −0.26 e Å^−3^

### 

Data collection: *SMART* (Bruker, 1998 ▸); cell refinement: *SAINT-Plus* (Bruker,1998 ▸); data reduction: *SAINT-Plus*; program(s) used to solve structure: *SHELXS97* (Sheldrick, 2008 ▸); program(s) used to refine structure: *SHELXL97* (Sheldrick, 2008 ▸); molecular graphics: *ORTEP-3 for Windows* (Farrugia, 2012 ▸) and *CAMERON* (Watkin *et al.*, 1996 ▸); software used to prepare material for publication: *WinGX* (Farrugia, 2012 ▸).

## Supplementary Material

Crystal structure: contains datablock(s) global, I. DOI: 10.1107/S2056989015018873/tk5392sup1.cif

Structure factors: contains datablock(s) I. DOI: 10.1107/S2056989015018873/tk5392Isup2.hkl

Supporting information file. DOI: 10.1107/S2056989015018873/tk5392Isup3.cml

**Fig. 1.**
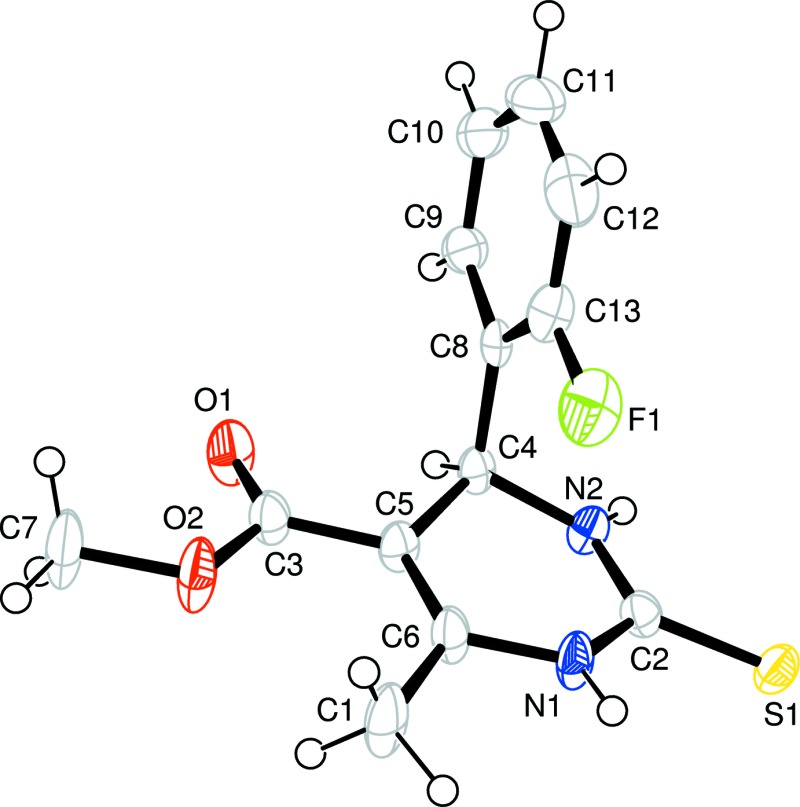
The molecular structure of the title compound with the atom-numbering scheme. Displacement ellipsoids are drawn at the 50% probability level. H atoms are presented as small spheres of arbitrary radius.

. DOI: 10.1107/S2056989015018873/tk5392fig1.tif
The mol­ecular structure of the title compound with the atom-numbering scheme. Displacement ellipsoids are drawn at the 50% probability level. H atoms are presented as small spheres of arbitrary radius.

**Fig. 2.**
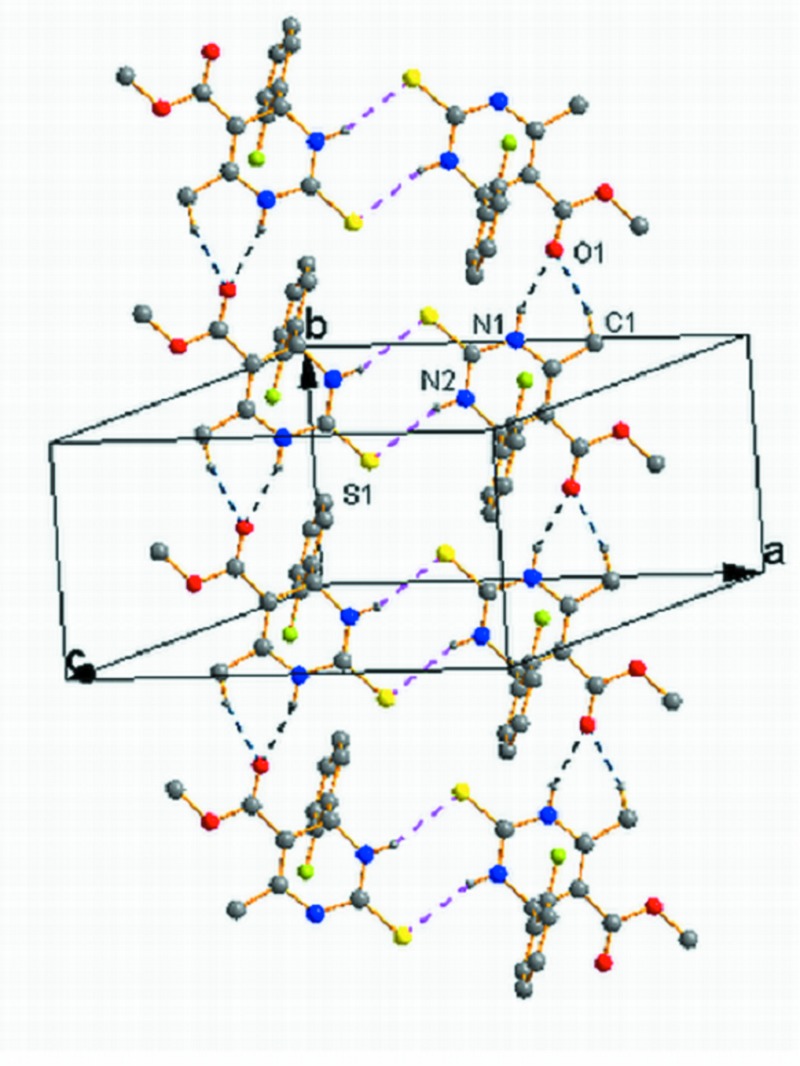
Unit cell packing of the title compound showing intermolecular C—H···O, N—H···O and N—H···S interactions as dotted lines. H atoms not involved in hydrogen bonding have been excluded.

. DOI: 10.1107/S2056989015018873/tk5392fig2.tif
Unit cell packing of the title compound showing inter­molecular C—H⋯O, N—H⋯O and N—H⋯S inter­actions as dotted lines. H atoms not involved in hydrogen bonding have been excluded.

CCDC reference: 1430034

Additional supporting information:  crystallographic information; 3D view; checkCIF report

## Figures and Tables

**Table 1:** Hydrogen-bond geometry (, ) *Cg* is the centroid of the C8C13 ring.

*D*H*A*	*D*H	H*A*	*D* *A*	*D*H*A*
N1H1O1^i^	0.88	2.14	2.977(4)	159
N2H2S1^ii^	0.88	2.55	3.386(2)	159
C1H1*B*O1^i^	0.98	2.52	3.262(5)	133
C10H10*Cg* ^iii^	0.95	2.86	3.648(2)	141

Symmetry codes: (i) 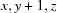; (ii) 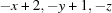; (iii) 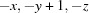.

## supplementary crystallographic information

### S1. Comment

In recent years, dihydropyrimidines (DHPMs) and their derivatives have attracted considerable attention in synthetic organic chemistry because of their wide range of biological activities (Kappe *et al.*, 2000), such as antibacterial, antiviral, antitumor and anti-inflammatory activities (Mayer *et al.*, 1999). The Biginelli reaction (Biginelli *et al.*, 1893), a one-pot condensation of aldehyde, acetoacetate and urea under strongly acidic conditions, is one of the most useful multicomponent reactions (MCRs), gaining increasing importance in organic and medicinal chemistry because of its capacity to generate multifunctionalized products including 3,4-dihydropyrimidin-2-ones, their thione analogs, and other related heterocyclic compounds. They are also noteworthy as calcium channel modulators (Kappe, 1998; Jauk *et al.*, 2000). The presence of a fluorine atom in the molecule can have profound and unexpected results on the biological activity of the compound (Guru Row, 1999; Yamazaki *et al.*, 2009). Herein, we report the crystal structure of the title compound. It is one of the analogue of our previously reported fluoro-DHPMs (Krishnamurthy & Begum, 2015a; Krishnamurthy & Begum, 2015b). The bond lengths and angles in the title compound are in good agreement with the corresponding bond distances and angles reported in closely related structures (Quin *et al.*, 2006).

In the title compound, Fig. 1, the 2-fluorobenzene ring at chiral carbon atom C4 is positioned axially and bisects the pyrimidine ring with a dihedral angle of 89.13 (4)°. The pyrimidine ring adopts a twist-boat conformation with atoms C4 and N1 displaced by 0.2739 (3) Å and 0.0938 (6) Å from the mean plane of the other four atoms (C5/C6/C2/N2) respectively. The carbonyl group of the exocyclic ester at C5 adopts a *trans* orientation with respect to C5=C6 double bond. The 2-fluorobenzene ring shows an *anti* periplanar conformation with respect to C4—H4 bond of the pyrimidine ring. The molecular structure is stabilized by intermolecular C1—H1B···O1 and N1—H1···O1 interactions generating bifurcated bonds from two donor atoms C1 and N1, to the same acceptor O1 to form an *R*^2^_2_(6) ring motif, which are in turn linked to form a molecular chain along crystallographic *b* axis. The packing is further stabilized by intermolecular N—H···S hydrogen bonds (N2—H2···S1) resulting in a centrosymmetric head to head dimer with graph set *R*^2^_2_(8) notation (Table 1; Fig. 2). In addition, the crystal structure is stabilized by C10—H10···*Cg* (*Cg* is the centroid of aryl ring C8—C13) interaction (Table 1).

### S2. Experimental

The title compound was synthesized by the reaction of 2-fluorobenzaldehyde (1.24 g, 10 mmol), methylacetoacetate (1.38 g, 12 mmol) and thiourea (1.14 g, 15 mmol) in 15 ml ethanol. The solution was refluxed for 6 h in the presence of concentrated hydrochloric acid as a catalyst. The reaction was monitored with TLC and the reaction medium was quenched in ice cold water. The precipitate obtained was filtered and dried. The compound was recrystallized from ethanol solvent by slow evaporation method, yielding colorless blocks suitable for X-ray diffraction studies (yield 72%; m.p. 476 K).

### S3. Refinement

The H atoms were placed at calculated positions in the riding-model approximation with N—H = 0.86 Å and C—H = 0.93–0.96 Å, and with *U*_iso_(H) = 1.5*U*_eq_(C) for methyl H atoms and *U*_iso_(H) = 1.2*U*_eq_(N, C) for the other hydrogen atoms.

### Crystal data

**Table:**

C_13_H_13_FN_2_O_2_S	*F*(000) = 584
*M**_r_* = 280.31	*D*_x_ = 1.425 Mg m^−^^3^
Monoclinic, *P*2_1_/*n*	Mo *K*α radiation, λ = 0.71073 Å
Hall symbol: -P 2yn	Cell parameters from 2296 reflections
*a* = 13.3298 (15) Å	θ = 3.0–25.0°
*b* = 7.1509 (8) Å	µ = 0.26 mm^−^^1^
*c* = 14.5703 (17) Å	*T* = 100 K
β = 109.854 (4)°	Block, colourless
*V* = 1306.3 (3) Å^3^	0.24 × 0.22 × 0.18 mm
*Z* = 4	

### Data collection

**Table:**

Bruker SMART APEX CCD diffractometer	2296 independent reflections
Radiation source: fine-focus sealed tube	1340 reflections with *I* > 2σ(*I*)
Graphite monochromator	*R*_int_ = 0.112
ω scans	θ_max_ = 25.0°, θ_min_ = 3.0°
Absorption correction: multi-scan (*SADABS*; Bruker, 1998)	*h* = −15→15
*T*_min_ = 0.955, *T*_max_ = 0.960	*k* = −8→8
9937 measured reflections	*l* = −15→17

### Refinement

**Table:**

Refinement on *F*^2^	Primary atom site location: structure-invariant direct methods
Least-squares matrix: full	Secondary atom site location: difference Fourier map
*R*[*F*^2^ > 2σ(*F*^2^)] = 0.064	Hydrogen site location: inferred from neighbouring sites
*wR*(*F*^2^) = 0.133	H-atom parameters constrained
*S* = 0.95	*w* = 1/[σ^2^(*F*_o_^2^) + (0.0421*P*)^2^ + 1.6067*P*] where *P* = (*F*_o_^2^ + 2*F*_c_^2^)/3
2296 reflections	(Δ/σ)_max_ < 0.001
174 parameters	Δρ_max_ = 0.42 e Å^−^^3^
0 restraints	Δρ_min_ = −0.26 e Å^−^^3^

### Special details

**Table:**

Geometry. All s.u.'s (except the s.u. in the dihedral angle between two l.s. planes) are estimated using the full covariance matrix. The cell s.u.'s are taken into account individually in the estimation of s.u.'s in distances, angles and torsion angles; correlations between s.u.'s in cell parameters are only used when they are defined by crystal symmetry. An approximate (isotropic) treatment of cell s.u.'s is used for estimating s.u.'s involving l.s. planes.
Refinement. Refinement of *F*^2^ against ALL reflections. The weighted *R*-factor *wR* and goodness of fit *S* are based on *F*^2^, conventional *R*-factors *R* are based on *F*, with *F* set to zero for negative *F*^2^. The threshold expression of *F*^2^ > 2σ(*F*^2^) is used only for calculating *R*-factors(gt) *etc*. and is not relevant to the choice of reflections for refinement. *R*-factors based on *F*^2^ are statistically about twice as large as those based on *F*, and *R*- factors based on ALL data will be even larger.

### Fractional atomic coordinates and isotropic or equivalent isotropic displacement parameters (Å^2^)

**Table:**

	*x*	*y*	*z*	*U*_iso_*/*U*_eq_	
S1	0.95357 (8)	0.77963 (14)	0.05365 (8)	0.0312 (3)	
O1	0.6807 (2)	0.0312 (4)	0.0894 (2)	0.0452 (9)	
O2	0.5913 (2)	0.2422 (3)	0.1440 (2)	0.0460 (8)	
N1	0.7803 (2)	0.6600 (4)	0.0856 (2)	0.0310 (9)	
H1	0.7685	0.7788	0.0941	0.037*	
N2	0.8688 (2)	0.4407 (4)	0.0289 (2)	0.0243 (8)	
H2	0.9267	0.4070	0.0170	0.029*	
F1	0.64843 (18)	0.5589 (3)	−0.10951 (18)	0.0491 (7)	
C1	0.6436 (4)	0.6093 (5)	0.1568 (4)	0.0500 (14)	
H1A	0.5696	0.6128	0.1121	0.075*	
H1B	0.6672	0.7365	0.1789	0.075*	
H1C	0.6481	0.5311	0.2133	0.075*	
C2	0.8628 (3)	0.6175 (5)	0.0542 (3)	0.0257 (9)	
C3	0.6629 (3)	0.1929 (5)	0.1031 (3)	0.0305 (10)	
C4	0.7879 (3)	0.2971 (5)	0.0189 (3)	0.0229 (9)	
H4	0.8262	0.1789	0.0469	0.027*	
C5	0.7196 (3)	0.3493 (5)	0.0792 (3)	0.0251 (9)	
C6	0.7138 (3)	0.5290 (5)	0.1050 (3)	0.0306 (10)	
C7	0.5324 (4)	0.0920 (6)	0.1694 (4)	0.0523 (14)	
H7A	0.4917	0.0232	0.1103	0.079*	
H7B	0.4833	0.1446	0.1997	0.079*	
H7C	0.5824	0.0068	0.2154	0.079*	
C8	0.7244 (3)	0.2591 (5)	−0.0870 (3)	0.0237 (9)	
C9	0.7339 (3)	0.0877 (6)	−0.1298 (3)	0.0323 (10)	
H9	0.7803	−0.0052	−0.0914	0.039*	
C10	0.6779 (3)	0.0514 (7)	−0.2255 (3)	0.0429 (12)	
H10	0.6851	−0.0666	−0.2525	0.052*	
C11	0.6117 (4)	0.1832 (8)	−0.2826 (3)	0.0550 (15)	
H11	0.5737	0.1571	−0.3494	0.066*	
C12	0.5999 (3)	0.3552 (8)	−0.2436 (3)	0.0509 (14)	
H12	0.5536	0.4478	−0.2824	0.061*	
C13	0.6574 (3)	0.3874 (6)	−0.1470 (3)	0.0343 (10)	

### Atomic displacement parameters (Å^2^)

**Table:**

	*U*^11^	*U*^22^	*U*^33^	*U*^12^	*U*^13^	*U*^23^
S1	0.0345 (6)	0.0216 (5)	0.0415 (6)	−0.0051 (5)	0.0183 (5)	−0.0047 (5)
O1	0.072 (2)	0.0159 (16)	0.070 (2)	0.0026 (15)	0.0524 (19)	−0.0029 (15)
O2	0.058 (2)	0.0187 (16)	0.085 (2)	−0.0037 (14)	0.0553 (19)	−0.0002 (15)
N1	0.043 (2)	0.0139 (17)	0.048 (2)	0.0005 (16)	0.0300 (19)	0.0032 (15)
N2	0.0213 (18)	0.0183 (18)	0.036 (2)	0.0019 (15)	0.0136 (15)	−0.0013 (15)
F1	0.0443 (16)	0.0364 (15)	0.0604 (17)	0.0111 (13)	0.0097 (13)	0.0185 (13)
C1	0.075 (4)	0.014 (2)	0.089 (4)	−0.003 (2)	0.065 (3)	−0.004 (2)
C2	0.030 (2)	0.024 (2)	0.026 (2)	0.0024 (19)	0.013 (2)	0.0033 (18)
C3	0.039 (3)	0.022 (2)	0.039 (2)	0.001 (2)	0.023 (2)	0.002 (2)
C4	0.027 (2)	0.0146 (19)	0.029 (2)	0.0040 (18)	0.0123 (18)	0.0056 (18)
C5	0.031 (2)	0.014 (2)	0.034 (2)	0.0049 (18)	0.017 (2)	0.0040 (17)
C6	0.041 (3)	0.019 (2)	0.042 (3)	0.001 (2)	0.027 (2)	0.0048 (19)
C7	0.067 (3)	0.024 (2)	0.095 (4)	−0.009 (2)	0.065 (3)	−0.002 (3)
C8	0.023 (2)	0.024 (2)	0.028 (2)	−0.0047 (19)	0.0144 (18)	0.0040 (19)
C9	0.031 (3)	0.038 (3)	0.033 (3)	−0.006 (2)	0.017 (2)	−0.006 (2)
C10	0.034 (3)	0.060 (3)	0.041 (3)	−0.019 (3)	0.022 (2)	−0.014 (3)
C11	0.045 (3)	0.090 (5)	0.030 (3)	−0.031 (3)	0.013 (3)	−0.012 (3)
C12	0.027 (3)	0.076 (4)	0.042 (3)	−0.008 (3)	0.000 (2)	0.024 (3)
C13	0.029 (2)	0.030 (3)	0.045 (3)	−0.003 (2)	0.015 (2)	0.002 (2)

### Geometric parameters (Å, º)

**Table:**

S1—C2	1.677 (4)	C4—C5	1.512 (5)
O1—C3	1.211 (4)	C4—H4	1.0000
O2—C3	1.332 (4)	C5—C6	1.348 (5)
O2—C7	1.451 (4)	C7—H7A	0.9800
N1—C2	1.362 (4)	C7—H7B	0.9800
N1—C6	1.383 (5)	C7—H7C	0.9800
N1—H1	0.8800	C8—C13	1.368 (5)
N2—C2	1.327 (4)	C8—C9	1.400 (5)
N2—C4	1.460 (4)	C9—C10	1.364 (5)
N2—H2	0.8800	C9—H9	0.9500
F1—C13	1.364 (5)	C10—C11	1.365 (7)
C1—C6	1.502 (5)	C10—H10	0.9500
C1—H1A	0.9800	C11—C12	1.386 (7)
C1—H1B	0.9800	C11—H11	0.9500
C1—H1C	0.9800	C12—C13	1.374 (6)
C3—C5	1.457 (5)	C12—H12	0.9500
C4—C8	1.511 (5)		
			
C3—O2—C7	116.8 (3)	C5—C6—N1	119.1 (3)
C2—N1—C6	124.4 (3)	C5—C6—C1	127.5 (4)
C2—N1—H1	117.8	N1—C6—C1	113.3 (3)
C6—N1—H1	117.8	O2—C7—H7A	109.5
C2—N2—C4	125.8 (3)	O2—C7—H7B	109.5
C2—N2—H2	117.1	H7A—C7—H7B	109.5
C4—N2—H2	117.1	O2—C7—H7C	109.5
C6—C1—H1A	109.5	H7A—C7—H7C	109.5
C6—C1—H1B	109.5	H7B—C7—H7C	109.5
H1A—C1—H1B	109.5	C13—C8—C9	116.1 (4)
C6—C1—H1C	109.5	C13—C8—C4	123.3 (3)
H1A—C1—H1C	109.5	C9—C8—C4	120.5 (3)
H1B—C1—H1C	109.5	C10—C9—C8	121.4 (4)
N2—C2—N1	116.0 (3)	C10—C9—H9	119.3
N2—C2—S1	123.1 (3)	C8—C9—H9	119.3
N1—C2—S1	120.9 (3)	C9—C10—C11	120.6 (5)
O1—C3—O2	122.4 (3)	C9—C10—H10	119.7
O1—C3—C5	123.2 (4)	C11—C10—H10	119.7
O2—C3—C5	114.3 (3)	C10—C11—C12	120.1 (4)
N2—C4—C8	111.5 (3)	C10—C11—H11	119.9
N2—C4—C5	109.8 (3)	C12—C11—H11	119.9
C8—C4—C5	113.5 (3)	C13—C12—C11	117.9 (4)
N2—C4—H4	107.2	C13—C12—H12	121.1
C8—C4—H4	107.2	C11—C12—H12	121.1
C5—C4—H4	107.2	F1—C13—C8	118.3 (4)
C6—C5—C3	125.5 (3)	F1—C13—C12	117.8 (4)
C6—C5—C4	120.1 (3)	C8—C13—C12	123.9 (4)
C3—C5—C4	114.4 (3)		
			
C4—N2—C2—N1	9.1 (5)	C4—C5—C6—C1	174.3 (4)
C4—N2—C2—S1	−173.8 (3)	C2—N1—C6—C5	−9.7 (6)
C6—N1—C2—N2	9.3 (5)	C2—N1—C6—C1	168.7 (4)
C6—N1—C2—S1	−167.8 (3)	N2—C4—C8—C13	−66.5 (4)
C7—O2—C3—O1	−1.7 (6)	C5—C4—C8—C13	58.1 (5)
C7—O2—C3—C5	−180.0 (4)	N2—C4—C8—C9	111.8 (4)
C2—N2—C4—C8	103.4 (4)	C5—C4—C8—C9	−123.5 (4)
C2—N2—C4—C5	−23.3 (5)	C13—C8—C9—C10	−1.0 (5)
O1—C3—C5—C6	−170.2 (4)	C4—C8—C9—C10	−179.4 (3)
O2—C3—C5—C6	8.1 (6)	C8—C9—C10—C11	0.8 (6)
O1—C3—C5—C4	10.8 (6)	C9—C10—C11—C12	−0.7 (6)
O2—C3—C5—C4	−170.9 (3)	C10—C11—C12—C13	0.6 (6)
N2—C4—C5—C6	21.7 (5)	C9—C8—C13—F1	−177.7 (3)
C8—C4—C5—C6	−103.9 (4)	C4—C8—C13—F1	0.7 (5)
N2—C4—C5—C3	−159.3 (3)	C9—C8—C13—C12	1.0 (6)
C8—C4—C5—C3	75.1 (4)	C4—C8—C13—C12	179.4 (4)
C3—C5—C6—N1	173.6 (4)	C11—C12—C13—F1	177.9 (4)
C4—C5—C6—N1	−7.5 (6)	C11—C12—C13—C8	−0.8 (6)
C3—C5—C6—C1	−4.6 (7)		

### Hydrogen-bond geometry (Å, º)

Cg is the centroid of the C8–C13 ring.

**Table:**

*D*—H···*A*	*D*—H	H···*A*	*D*···*A*	*D*—H···*A*
N1—H1···O1^i^	0.88	2.14	2.977 (4)	159
N2—H2···S1^ii^	0.88	2.55	3.386 (2)	159
C1—H1*B*···O1^i^	0.98	2.52	3.262 (5)	133
C10—H10···*Cg*^iii^	0.95	2.86	3.648 (2)	141

Symmetry codes: (i) *x*, *y*+1, *z*; (ii) −*x*+2, −*y*+1, −*z*; (iii) −*x*, −*y*+1, −*z*.
