# Supplementary figures and images for: Crystal structure of methyl 4-(2-fluoro­phenyl)-6-methyl-2-sulfanylidene-1,2,3,4-tetra­hydro­pyrimidine-5-carb­oxy­late
Source: Acta Crystallogr E Crystallogr Commun. 2015 Oct 14;71(Pt 11):o838–9. doi: 10.1107/S2056989015018873 (PMC4645072; doi:10.1107/S2056989015018873)

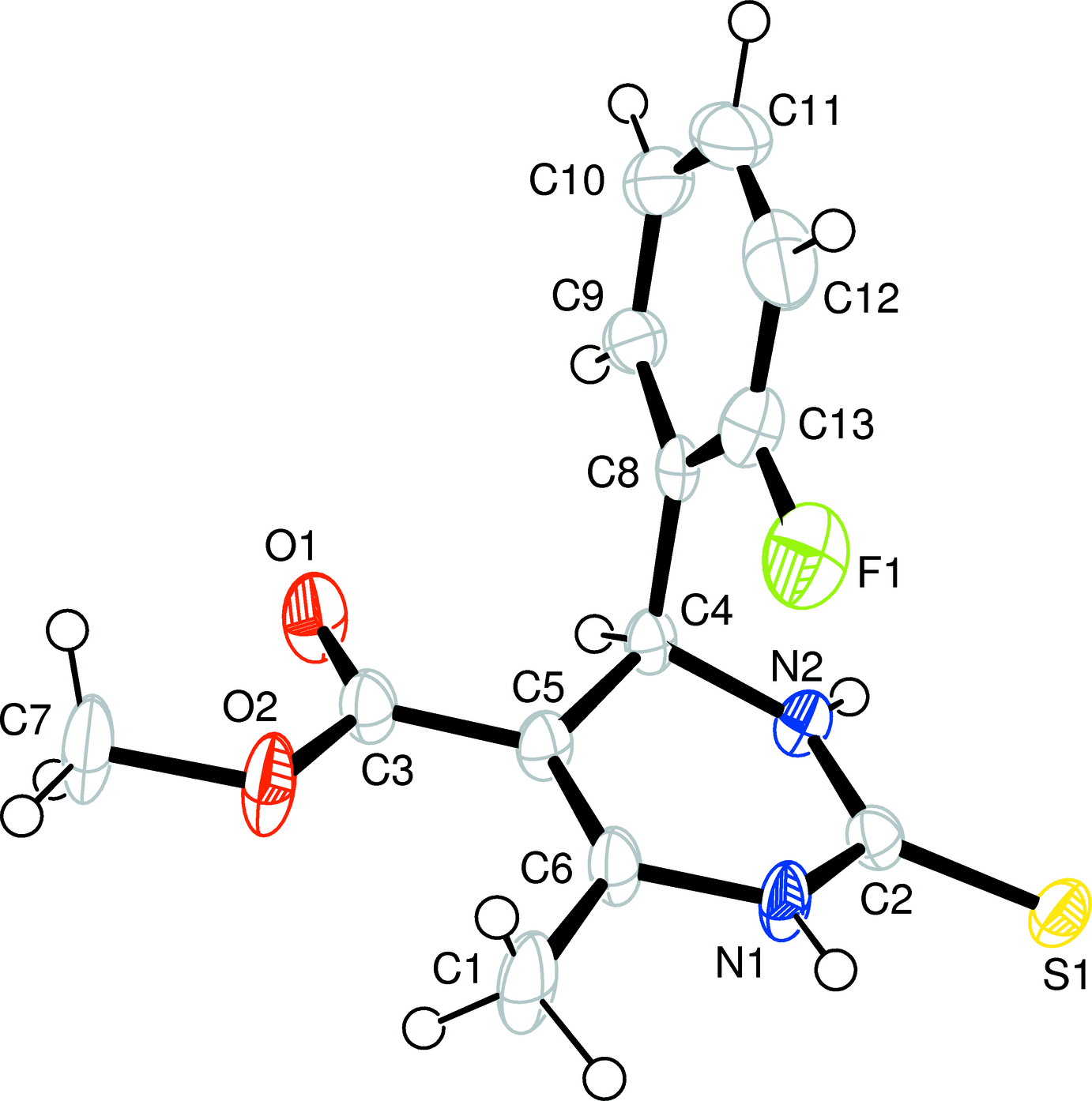

Supplement: Supplementary file 4 [file e-71-0o838-fig1.tif]

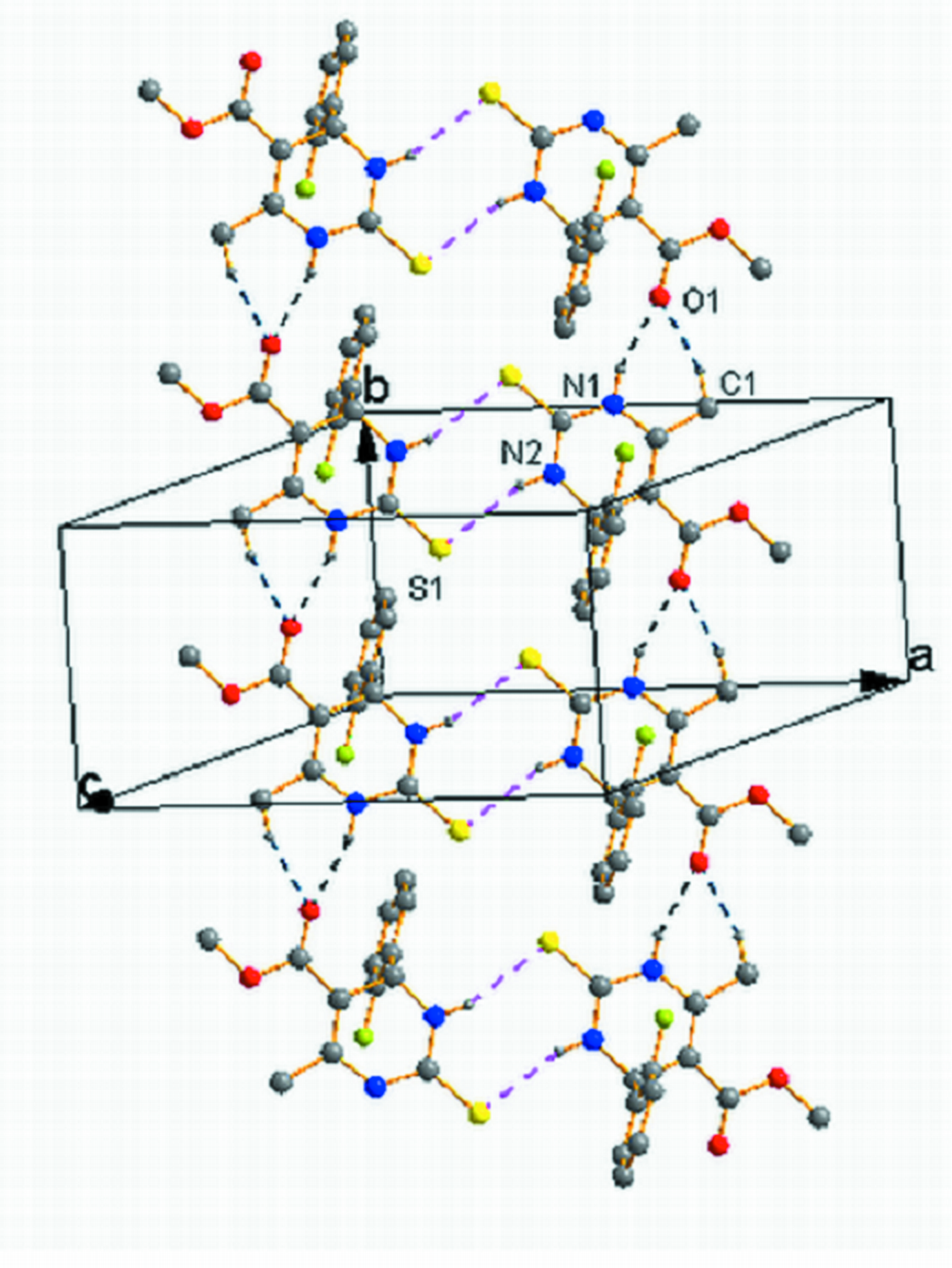

Supplement: Supplementary file 5 [file e-71-0o838-fig2.tif]
